# Inhibition of SLPI ameliorates disease activity in experimental autoimmune encephalomyelitis

**DOI:** 10.1186/1471-2202-13-30

**Published:** 2012-03-21

**Authors:** André Michael Müller, Esther Jun, Hana Conlon, Saud Ahmed Sadiq

**Affiliations:** 1 Multiple Sclerosis Research Center of New York, 521 W 57th Street, 4th floor, New York, NY, 10019, USA

**Keywords:** SLPI, EAE, TGF-beta, Multiple sclerosis

## Abstract

**Background:**

The secretory leukocyte protease inhibitor (SLPI) exerts wide ranging effects on inflammatory pathways and is upregulated in EAE but the biological role of SLPI in EAE, an animal model of multiple sclerosis is unknown

**Methods:**

To investigate the pathophysiological effects of SLPI within EAE, we induced SLPI-neutralizing antibodies in mice and rats to determine the clinical severity of the disease. In addition we studied the effects of SLPI on the anti-inflammatory cytokine TGF-β.

**Results:**

The induction of SLPI neutralizing antibodies resulted in a milder disease course in mouse and rat EAE. SLPI neutralization was associated with increased serum levels of TGF-β and increased numbers of FoxP3+ CD4+ T cells in lymph nodes. *In vitro*, the addition of SLPI significantly decreased the number of functional FoxP3+ CD25^hi ^CD4+ regulatory T cells in cultures of naive human CD4+ T cells. Adding recombinant TGF-β to SLPI-treated human T cell cultures neutralized SLPI's inhibitory effect on regulatory T cell differentiation.

**Conclusion:**

In EAE, SLPI exerts potent pro-inflammatory actions by modulation of T-cell activity and its neutralization may be beneficial for the disease.

## Background

The secretory leukocyte protease inhibitor (SLPI) is an 11.7 kDa protein originally identified in bodily secretions such as saliva, seminal fluid, and in the mucus of cervical, nasal and bronchial passages [1]. It was later found in neutrophils, peritoneal macrophages, astrocytes and neurons [2,3] as well as in activated regulatory T cells [4], and was shown to be strongly upregulated in the CNS as a consequence of ischemic stroke [2], spinal cord injury [5] and experimental autoimmune encephalomyelitis (EAE) [3]. SLPI is a potent inhibitor of leukocyte serine proteases, including elastase and cathepsin G from neutrophils, chymase and tryptase from mast cells, and trypsin and chymotrypsin from pancreatic acinar cells [6]. In addition, SLPI suppresses bacterial growth [7], inhibits HIV-1 infection of macrophages [8] and exerts anti-inflammatory functions in macrophages, neutrophils and B cells by inhibition of IκBα degradation [9,10]. Finally, SLPI diminishes inflammatory gene expression and inflammatory cell accumulation after hepatic and lung injuries [11], is neuroprotective in an ischemic stroke model [2] and suppresses the expression of matrix metalloproteinases by stimulated monocytes [12].

Mice deficient in SLPI show impaired cutaneous wound healing with increased inflammation. Additionally, an increased TGF-β activity was found in these mice, likely due to an increased proteolytic activation of latent TGF-β in SLPI-deficient animals [13]. SLPI-mediated suppression of TGF-β expression by human endometrial cells [14] and SLPI's inhibition on the induction of regulatory T cell differentiation by elastase [15] provide corroborating evidence that it has prominent proinflammatory properties.

We investigated whether the effects of SLPI on the immune system may have implications in diseases characterized pathologically by inflammation as a result of autoimmune mechanisms such as multiple sclerosis (MS). Indeed SLPI is known to be markedly upregulated in a rat model of the disease called experimental autoimmune encephalomyelitis [3]. EAE can be induced by immunization with myelin proteins which result in auto-reactive CD4+ T cells to react with myelin and cause concomitant clinical disease. The inflammatory lesions in EAE strongly mimic the acute lesion in MS [16,17]. Based on all these findings our studies aimed to determine the role of SLPI in the pathogenesis of EAE in SJL/J mice and DA rats and to study the impact of SLPI on TGF-β activity.

## Methods

### Animals

Female dark agouti (DA) rats, 6-8-weeks old were purchased from Harlan Laboratories (Indianapolis, IN), and female SJL/J mice, 6-8-weeks old, were purchased from the Jackson Laboratory (Bar Harbor, ME). Animals were housed in the animal facility of Roosevelt Hospital (New York, NY) and were 8-10-week old when used for experiments. All procedures were conducted according to protocols approved by the IACUC committee of Roosevelt Hospital.

### Induction and clinical evaluation of EAE

For active EAE induction, SJL/J mice were immunized with 200 μL of a suspension containing 200 μg of murine PLP peptide (aa 139-151 (HSLGKWLGHPDKF), Pepceuticals, Leicestershire, UK), and an equal volume of CFA supplemented with 500 μg H37RA by subcutaneous injection to generate PLP-specific encephalitogenic lymph node cells.

In order to induce the adoptive transfer EAE (at-EAE) in SJL/J mice, lymph node cells were harvested 10 days after PLP-immunization and restimulated *in vitro *for four days with 10 μg/mL PLP-peptide. Naive female SJL/J mice were injected intraperitoneally (*ip*) with 1.5 × 10^7 ^preactivated PLP-specific LNC for at-EAE induction.

To induce active EAE in DA rats, animals were immunized subcutaneously at the base of the tail with 65 μg MOG1-125 emulsified in complete Freund's adjuvant (CFA) supplemented with 400 μg of heat-inactivated *Mycobacterium tuberculosis *(H37Ra) (DIFCO Laboratories, Detroit, MI) in a total volume of 200 μL.

Animal weight and clinical score were recorded daily (0 = healthy, 1 = limp tail, 2 = partial hind limb weakness and/or ataxia, 3 = complete paralysis of at least one hind limb, 4 = severe forelimb weakness, 5 = moribund or dead). The mean cumulative score for a treatment group was calculated as the sum of the daily scores of all animals from day zero until the end of the experiment divided by the number of animals in the respective group.

### Protein vaccination

SJL/J mice and DA rats were immunized i.p. with 100 μL of a solution containing 10 μg of SLPI (R&D Systems), respectively, mixed with 30 μL of the adjuvant aluminum hydroxide (Aluhydrogel; Brenntag Biosector, Frederikssund, Denmark). Vaccinations were performed twice with an interval of 3 weeks as previously described [18]. Control animals were injected with OVA peptide (aa 323-339 ANASPEC (Fremont, CA)) and aluminum hydroxide. After the second vaccination, animals rested 6 to 8 weeks before EAE was induced by adoptive transfer of encephalitogenic lymph node cells (LNC) or MOG-protein immunization, respectively.

### Enzyme-linked immunosorbent assay (ELISA) for anti-SLPI antibody detection

A direct ELISA assay was used to confirm SLPI-specific IgG titers in sera from mice that had received the second vaccination two weeks before. 96-well ELISA plates (Nunc, Roskilde, Denmark) were coated overnight at 4°C with recombinant SLPI protein (R&D Systems (Minneapolis, MN)) at concentrations of 100 ng/well. Plates were washed with 0.1% PBS/Tween-20. Sera from SLPI protein-vaccinated or OVA-vaccinated mice or rats were then added in serial dilutions from 1:30 to 1:65610, and the plates were incubated for 1 h at RT. After washing, goat anti-mouse IgG conjugated to alkaline phosphatase or respectively goat anti-rat IgG conjugated to alkaline phosphatase (both from Sigma-Aldrich, St. Louis, MO) were used as secondary antibodies with p-nitrophenylphosphate (p-NPP; Sigma-Aldrich) as a substrate.

### Confirmation of SLPI neutralization by serum SLPI antibodies

IgG antibodies were purified from mice sera by utilizing the MabTrap™ kit (Amersham Biosciences, Piscataway, NJ) according to the manufacturer's protocol. Antibody titers to SLPI and OVA were confirmed by an ELISA assay as described above.

U937 cells (ATCC; Manassas, Virginia) were cultured at a density of 1 × 10^5 ^cells/200 μL RPMI medium in a 96-well plate with LPS (100 ng/mL), 4 μg/mL SLPI and/or 40 μg/mL of purified total IgG from mice vaccinated with either SLPI or OVA-peptide. After 2 h the cells were harvested, and RNA was isolated using the RNeasy Plus Mini Kit (Qiagen). The IL-8 expression was quantified using IL-8 specific Taqman probes (Hs99999034_m1, Applied Biosystems, Framingham, MA) and normalized to respective 18S rRNA quantities also determined by using Taqman probes (Applied Biosystems).

### SLPI administration

Recombinant SLPI (R&D Systems, Minneapolis, MN) was administered *ip *to female SJL/J mice for the first sixteen days after disease induction The experimental SLPI group of mice was injected with SLPI (100 μL with a SLPI concentration of 3.3 μg/mL in 0.9% saline), and the control group of mice received 100 μL of 0.9% saline instead. All mice received the injection *ip *three times a day.

### Quantification of TGF-β in serum samples

A direct ELISA assay was used to determine TGF-β levels in serum samples isolated from mice on day 37 after EAE induction and from DA rats at day 14 after EAE induction using the Mouse/Rat/Porcine/Canine TGF-β1 immunoassay (R&D Systems). Total TGF-β quantities were measured by incubating serum samples with 1 M hydrogen chloride which activates latent TGF-β. Activated TGF-β serum samples were analyzed without the addition of HCl.

### Isolation and cultivation of naive human CD4+ T cells

White blood cells were extracted from peripheral blood using CPT BD Vacutainer tubes (BD Biosciences). Naïve human CD4+ T cells were purified according to the Naive CD4^+ ^T Cell Isolation Kit II instructions (Miltenyi Biotec, Bergisch Gladbach, Germany).

100,000 Naïve human CD4+ T cells per 0.2 mL of RPMI were cultivated in U bottom-shaped 96-well plates. Cells were stimulated with αCD3- and αCD28-microbeads (Dynabeads^® ^Human T-Activator CD3/CD28, Invitrogen) in serum-free "Stem Line T cell expansion medium" (Sigma Aldrich).

### Determination of TGF-β expression by U937 cells

U937 cells (ATCC; Manassas, Virginia) were cultured at a density of 1 × 10^5 ^cells/200 μL RPMI medium in a 96-well plate with or without 500 ng/ml SLPI (R&D Systems). After 16 h the cells were harvested, and RNA was isolated using the RNeasy Plus Mini Kit (Qiagen). The TGF-β expression was quantified using TGF-β specific Taqman probes (Hs00998133_m1, Applied Biosystems) and normalized to respective 18S rRNA quantities also determined by using Taqman probes (Applied Biosystems).

### Flow cytometry

Flow cytometric analyses were carried out with a FACSARIA flow cytometer (BD Biosciences). After staining the cell surfaces, the cells were washed, fixed and permeabilized with the BD Cytofix/Cytoperm-Kit (BD Biosciences) and stained for intracellular molecules. The following antibodies were used for murine cells: Pacific Blue anti-CD4 (RM4-5) (BD Bioscience), PE anti IL-17A (clone eBio17B7) and APC anti-IFN-g (clone XMG1.2) (both from eBioscience, San Diego, CA) and with AlexaFluor647 anti-FoxP3 ((clone 259D) BioLegend, San Diego, CA). Human cells were stained with eFluor450 anti-CD4 (clone OKT4), PE anti-IFN-g (clone 4S.B3), FITC anti-IL-17 (clone eBio64DEC17), PE anti CD25 (clone BC96) (all four from eBioscience) as well as with AlexaFluor647 anti-FoxP3 (clone 259D) or AlexaFluor647 isotype antibody (AlexaFluor 647 mouse IgG1, κ, BioLegend).

To detect the proliferation of stimulated naïve human CD4+ T cells, cells were stained with 2.5 μM CFSE using the CellTrace™ CFSE Cell Proliferation Kit (Invitrogen, Carlsbad, CA) according to the manufacturer's instructions. The number of divisions was determined by counting the number of peaks. For the detection of intracellular IL-17 and IFN-g in CFSE stained cells, Alexa Fluor700 anti-IL-17a (clone N49-653, BD Biosciences) and PE-Cy7 anti-IFN-g (clone 4S.B3, BioLegend) antibodies were used. Additionally, antibodies of the same isotype as the FoxP3 antibody (AlexaFluor 647 mouse IgG1, κ, BioLegend) were used for staining.

### Statistical analyses

Statistical analyses were performed using SigmaStat 3.0 (IBM, Somers, NY). Comparisons of groups of normal-distributed data were done by Student's *t*-test or an ANOVA analysis. For non-normally distributed data from the EAE experiments the Mann-Whitney Rank Sum test was used for comparison. Error bars represent standard deviations unless otherwise indicated.

## Results

### Vaccination of SJL/J mice with SLPI protein induces SLPI-specific neutralizing antibodies

**Figure 1:**
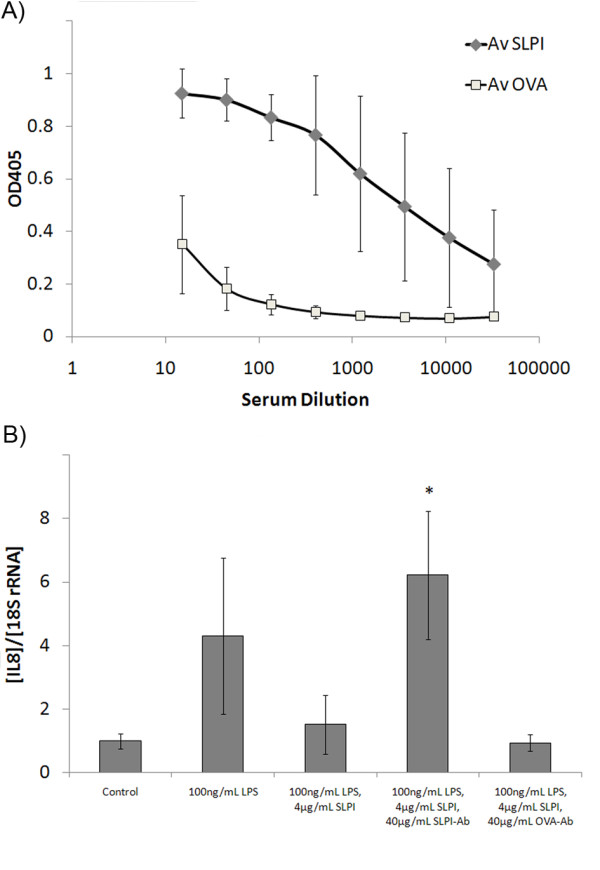
**SLPI protein vaccination induces neutralizing SLPI-specific antibodies**. **A**: Mouse sera collected 14 days after the second vaccination with SLPI- or OVA-protein were analyzed by ELISA for the presence of SLPI-specific antibodies, as described in Materials and Methods. IgG titers representing the average of samples from six different mice were compared. **B**: U937 monocytic cells were incubated with 4 μg/mL SLPI with either 40 μg/mL IgG antibodies purified from sera of SLPI-vaccinated mice or from OVA-vaccinated mice and stimulated for 2 h with 100 ng/mL LPS. The IL-8 expression was quantified by Real Time-PCR. Results are shown as mean ± standard deviation of three samples. The differences between LPS-stimulated cells and control cells, between SLPI and LPS-treated cells and just LPS treated cells, between LPS- and SLPI-treated cells and cells treated with SLPI, LPS and SLPI-IgG and between SLPI, LPS and SLPI-IgG and SLPI, LPS and OVA-IgG treated U937 cells were statistically significant at p < 0.05 (One-Way ANOVA and Holm-Sidak test)

Previously, we reported that SLPI is strongly induced within the spinal cord during the disease course of the MOG-induced EAE of DA rats [3]. To further address this observation, we induced neutralizing SLPI antibodies *in vivo *in order to neutralize SLPI and to assess its function during CNS inflammation. In particular, we immunized female SJL/J mice with human recombinant SLPI protein and control mice with ovalbumin peptide (OVA). Vaccinations were performed twice with an interval of three weeks and resulted in high and reproducible antibody titers for SLPI (Figure 1A).

To confirm that the induced SLPI-specific antibodies were able to neutralize SLPI's activity, we determined if they were able to prevent the SLPI-mediated inhibition of the LPS-induced production of IL-8 by the human monocytic cell line U937 [9]. We found that, serum IgG from SLPI-vaccinated mice neutralized SLPI's inhibitory effect on the LPS-induced IL-8 expression. In contrast, serum IgG isolated from control OVA-vaccinated mice had no impact on the inhibition of the LPS-mediated induction of IL-8 by SLPI (Figure 1B). These data confirmed that protein vaccination with recombinant SLPI leads to the generation of SLPI-specific antibodies that neutralize biological effects of soluble SLPI *in vitro*.

### SLPI blockade is protective in passive EAE of female SJL/J mice and in active EAE of DA rats

To investigate the role of SLPI *in vivo*, we examined the consequences of its neutralization in the passive EAE of SJL/J mice. For the induction of adoptive transfer EAE, donor SJL/J mice were immunized with PLP peptide. Their lymph node cells (LNC) were harvested 10 days later and restimulated *in vitro *for four days with PLP. 1.5 × 10^7 ^LNC were subsequently transferred into mice which had been immunized with OVA- or SLPI-protein two months beforehand.

**Figure 2:**
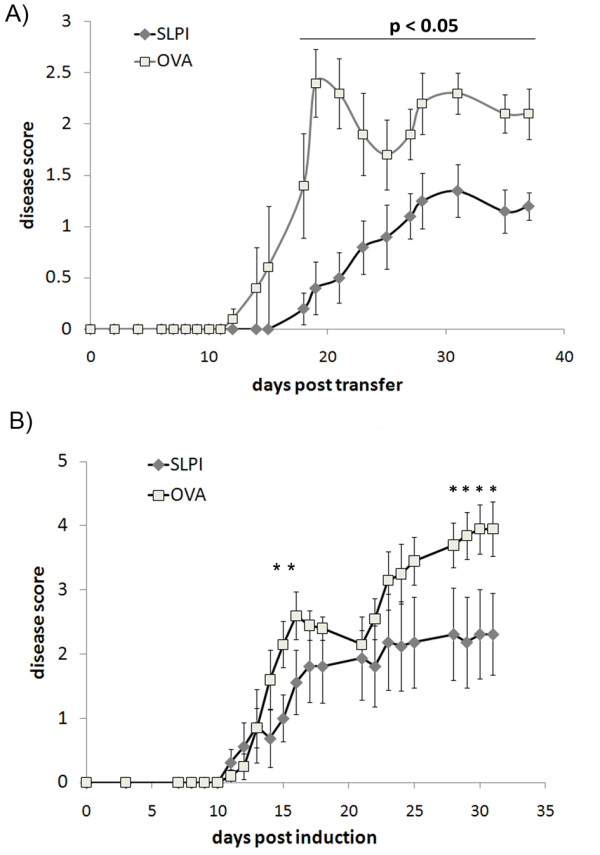
**SLPI protein vaccination protects against adoptive transfer EAE in mice and active EAE in rats**. **A**: SJL/J mice were vaccinated twice with SLPI or OVA and aluminum hydroxide. Six weeks after the last vaccination, EAE was induced by *i.p*. injection of encephalitogenic PLP specific LNC. Data are representative of two independent experiments and are shown as mean ± S.E. of the mean. The differences between the SLPI and the control group were statistically significant throughout the whole experiment after day 18 (Mann- Whitney rank sum test, p < 0.05). **B**: DA rats were vaccinated twice with SLPI- or OVA-protein and aluminum hydroxide. Six weeks after the last vaccination, EAE was induced by immunization with recombinant MOG protein. Data are representative of two experiments and are shown as mean ± S.E. of the mean. The differences between the SLPI and the control group were statistically significant on days 15 and 16 after disease induction as well as after day 25 (Mann-Whitney rank sum test, *, p < 0.05)

Mice vaccinated with SLPI protein developed a significantly less severe EAE disease course than the OVA-immunized animals (Figure 2A, Table 1) correlating with a significantly delayed disease onset in SLPI immunized mice compared to OVA-immunized mice. This finding suggests that the neutralization of SLPI by the induction of SLPI-specific antibodies had an ameliorative effect on the clinical course of EAE.

**Table 1:** Clinical parameters of EAE experiments in SJL/J mice and DA rats (*, p < 0

	Incidence Mortality		Disease onset [d] ± StdDev	Cumulative score ± StdDev	Mean maximal score ± StdDev
**SLPI immunization in passive EAE of SJL/J mice**					

SLPI-immunized mice	10/10	0/10	23.0 ± 4.6 *	8.9 ± 4.6 *	2 ± 0.5

OVA-immunized mice	7/7	0/7	17 ± 2.8	21.4 ± 7.5	2.6 ± 0.7

**SLPI immunization in active EAE of DA rats**					

SLPI-immunized rats	7/8	1/8	20.9 ± 10.8	21.2 ± 19.3	2.8 ± 2.0 *

OVA-immunized rats	8/8	3/8	13.8 ± 1.8	30.7 ± 9.3	4.5 ± 0.8

**SLPI administration in active EAE of SJL/J mice**					

SLPI-treated mice	6/7	1/7	11.1 ± 2.4 *	29.7 ± 15.3	3.1 ± 1.5

saline-treated mice	5/5	0/5	15.2 ± 4.4	19.8 ± 5.7	2.8 ± 0.4

We next studied the impact of SLPI neutralization on the active MOG-induced EAE of DA rats, because we previously showed that SLPI is substantially upregulated in the spinal cord in this model [3]. Rats were vaccinated in the same manner as the SJL mice with SLPI- or OVA-protein. Protein vaccination of rats also induced specific antibody titers for SLPI and OVA, respectively, as measured by ELISA (Additional file 1: Figure S1). While the onset of clinical disease after immunization with MOG 1-125 was not significantly delayed in SLPI-vaccinated rats, they developed a significantly milder disease course than mice vaccinated with OVA protein (Figure 2B, Table 1). Thus, the induction of SLPI-neutralizing antibodies decreases EAE disease severity in two species, irrespective of the autoantigen used for disease induction.

In order to corroborate SLPI's pathogenic role in EAE, we administered recombinant SLPI to SJL/J mice which have been immunized with PLP-peptide for active EAE induction. The SLPI group was injected intraperitoneally three times a day for sixteen days with SLPI (100 μL with a SLPI concentration of 3.3 μg/mL in 0.9% saline), control mice received 100 μL of 0.9% saline instead. SLPI treated mice developed disease signs earlier than the control mice and had a more severe disease course (Additional file 2: Figure S2A, Table 1). After stopping the administration of SLPI, the SLPI-treated animals became similar to the control mice. This might be explained by the limited half-life of SLPI *in vivo *[19]. The observed increase of disease symptoms of SLPI-treated mice after day 16 could also have been caused by the induction of SLPI antibodies, because we found high serum SLPI antibody titers in SLPI-immunized mice at day 30 after EAE induction (Additional file 2: Figure 2B).

### SLPI has an inhibitory effect on TGF-β *in vivo *and *in vitro*

SLPI is known to be an inhibitor of the anti-inflammatory molecule TGF-β by two different mechanisms: suppressing the expression of TGF-β [14] and interfering with TGF-β's proteolytic activation [13]. It was also described to interfere with the differentiation of TGF-β producing regulatory T cells induced by neutrophilic elastase [15].

**Figure 3:**
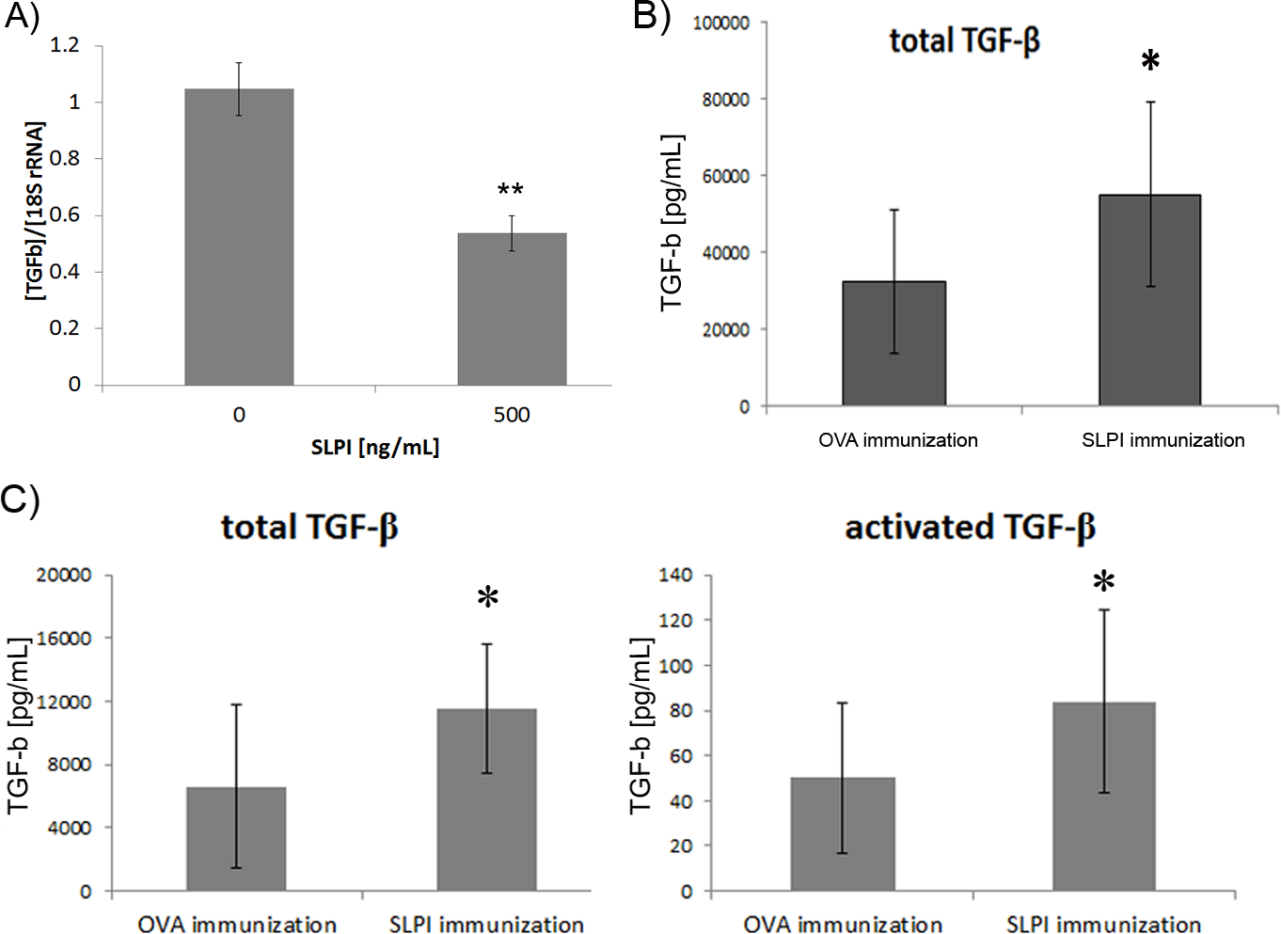
**SLPI has an inhibitory effect on TGF-β**. **A**: 100,000 U937 cells were incubated for 16 h in 0.2 mL RPMI medium with or without SLPI. TGF-β expression was quantified by RealTime-PCR (**, p < 0.01 (Student's *t*-test)) **B**: Blood was drawn from SLPI- and OVA-immunized mice at the end of the experiment shown in Figure 2A. Total TGF-β was quantified after activation of latent TGF-β in the samples by hydrogen chloride treatment. Differences between samples from SLPI-immunized and OVA-mice immunized were tested for their statistical significance using a Student's *t*-test. (*, *p *< 0.05) **C**: Blood was drawn from SLPI- and OVA-immunized rats at day 14 after EAE induction. Total TGF-β has been quantified after activation of latent TGF-β in the samples by hydrogen chloride treatment. Activated TGF-β levels were determined by skipping the activation step. Differences of total and activated TGF-β levels between samples isolated from SLPI-immunized and OVA-immunized rats were tested for their statistical significance using a Student's *t*-test. (*, p < 0.05)

Firstly, we incubated U937 cells U937 for 16 h with 500 ng/mL of recombinant SLPI, a SLPI concentration previously determined to have the greatest impact on the differentiation of neural stem cells [3]. Interestingly, SLPI strongly suppressed the expression of TGF-β quantified by RealTime-PCR (Figure 3A).

Next, we quantified TGF-β in sera obtained from SLPI- and OVA-immunized mice at the end of the experiment shown in Figure 2A and in serum samples isolated from rats at day 14 after EAE induction. Total TGF-β serum levels were significantly higher in SLPI-immunized than in OVA-immunized mice (Figure 3B). SLPI-immunized rats had significantly increased total and activated TGF-β level compared to OVA-immunized rats on day 14 after EAE induction (Figure 3C) suggesting that SLPI inhibits TGF-β production *in vivo*.

### SLPI inhibits differentiation of regulatory T cells

**Figure 4:**
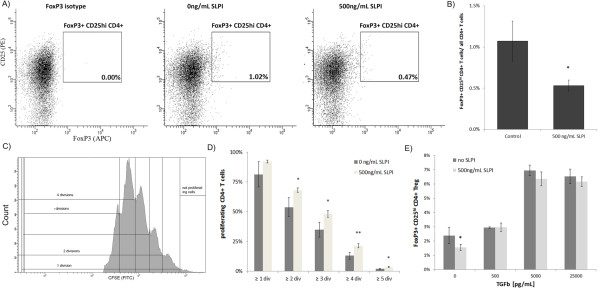
**SLPI reduces generation of regulatory T cells *in vitro***. **A**: Human naïve CD4+ T cells were stimulated with anti-CD3- and anti-CD28-coated beads in serum-free medium in the absence or presence of 500 ng/mL SLPI. After six days, cells were analyzed by flow cytometry for CD4 (DAPI channel (not shown)), CD25 (PE channel) and FoxP3 (APC channel). Left side: Dot blot representing staining with an antibody of the same isotype as the FoxP3 antibody (AlexaFluor 647 mouse IgG1, k, BioLegend), middle: representative dot blot of control treated naïve human CD4+ T cell cultures, right side: representative dot blot of T cell cultures incubated with 500 ng/mL SLPI. **B**: Column chart shows the average concentration of FoxP3+ CD25^hi ^CD4+ T cells in CD4+ T cell cultures. p = 0.011 comparing FoxP3+ CD25^hi ^CD4+ T cells incubated with and without 500 ng/mL SLPI. **C**: Human naïve CD4+ T cells were stimulated with anti-CD3- and anti-CD28-coated beads in RPMI medium in the absence or presence of 500 ng/mL SLPI. After four days, the cells were cocultured at a ratio of 1:5 with naïve CFSE labeled CD4+ T cells from the same donor. The CFSE profiles of the labeled cells were determined after 4 days. The number of divisions was determined by counting the number of peaks. Representative histogram of CSFE labeled T cells. **D**: Bars represent the proportion of CFSE labeled CD4+ T cells which proliferated and the total number of CFSE labeled cells. For every number of divisions, the differences between cell cultures containing SLPI-treated T cells and control treated T cells were tested for their statistical significance using a Suet' *t*-test (*, p < 0.05; **, p < 0.01). **E**: Human naïve CD4+ T cells were stimulated with anti-CD3- and anti-CD28-coated beads in RPMI medium in the absence or presence of 500 ng/mL SLPI and various concentrations of TGF-α. After four days, the cells were analyzed by flow cytometry for CD4 (DAPI channel), CD25 (PE channel) and FoxP3 (APC channel). The columns represent the average density of FoxP3+ CD4+ T cells in the *in vitro *cultures. *: p < 0.05 comparing FoxP3+ CD4 T cells incubated with and without SLPI

As TGF-β favors the generation of regulatory T cells, we investigated if SLPI interferes directly with the differentiation of regulatory T cells. Naïve human CD4+ T cells isolated from human blood were incubated for six days in serum-free medium in the presence or absence of 500 ng/mL SLPI protein. We detected fewer FoxP3+ CD25^hi ^CD4+ regulatory T cells in those T cell cultures treated with recombinant SLPI protein (Figures 4A and 4B) indicating that the SLPI decreases the generation of regulatory T cells. By contrast there were no differences in the number of Th1 and Th17 cells between SLPI treated- and control treated T cell cultures; no Th17 cells were detectable and less than 1% of the CD4+ T cells were Th1 cells (Additional file 3: Figure S3).

We next determined whether the SLPI-mediated decrease of CD4+ FoxP3+ T cells correlated with a reduced regulatory activity of SLPI-treated naïve human CD4+ T cells. Human naïve CD4+ T cells were stimulated with anti CD3- and CD28-antibody-coated beads with or without 500 ng/mL of SLPI as previously described. After four days, cells were further cultured at various ratios with fresh naïve CD4+ T cells isolated from the same donor and stained with CFSE. FACS analyses revealed that CSFE labeled CD4+ T cells cocultured with SLPI treated T cells for four days showed significantly more proliferation than cells cocultured with control treated T cells (Figures 4C and 4D) confirming that SLPI interferes with the regulatory activity of T cell cultures.

To validate that the observed effect on regulatory activity is mediated by suppression of TGF- β, naïve SLPI-treated CD4+ T cell cultures were supplemented with active human TGF-β (R&D Systems) and incubated for three days. The addition of only 500 pg/mL of TGF- β neutralized the effect of SLPI on the differentiation on FoxP3+ regulatory T cells completely (Figure 4E) corroborating that SLPI is interfering with the differentiation of regulatory T cells by inhibiting the activation of TGF- β.

**Figure 5:**
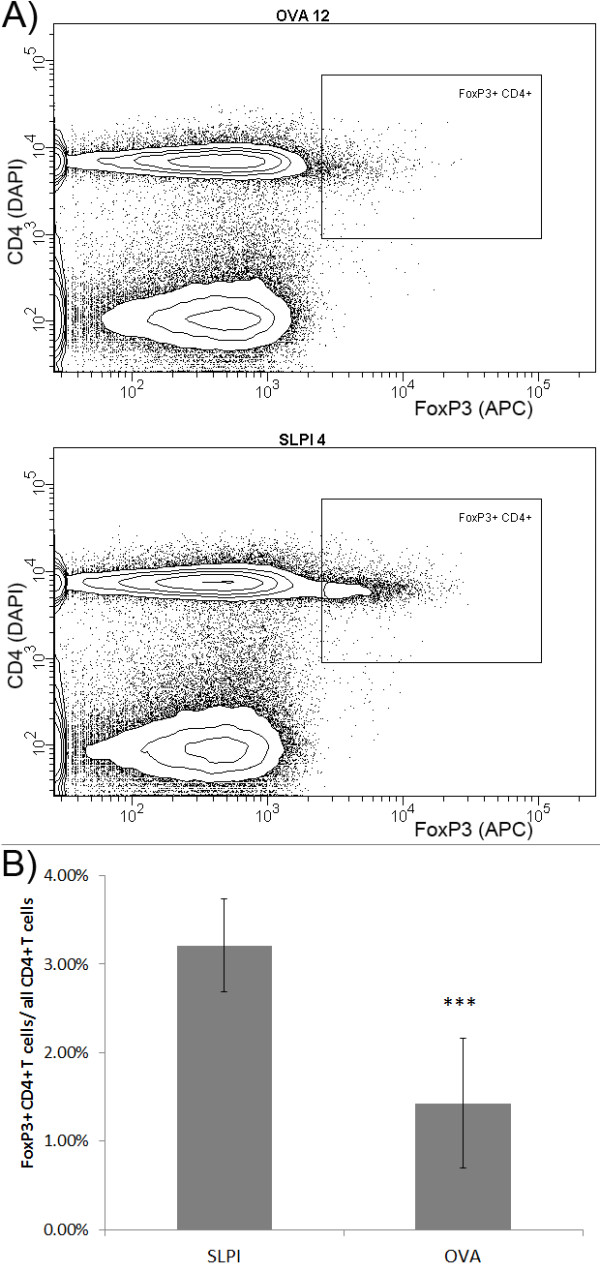
**SLPI protein immunization reduces number of FoxP3+ CD4+ T cells in lymph nodes of *in vivo***. **A**: 10 OVA- and 9 SLPI-immunized SJL/J were sacrificed 12 days after passive EAE induction. LNC were extracted and analyzed by flow cytometry for CD4 (DAPI channel) and FoxP3 (APC channel). Result from a representative OVA-immunized mouse is shown in the upper diagram and from a SLPI-immunized mouse in the lower diagram. **B**: Column chart represents the average ratio of FoxP3+ CD4+ and total number of CD4+ T cells in inguinal lymph nodes of SLPI- and OVA-immunized mice on day 12 after passive EAE induction (***, p < 0.001, Student's *t*-test)

We also addressed the *in vivo *influence of SLPI on regulatory T cell differentiation. In particular, we examined if the neutralization of SLPI decreases the number of these anti-inflammatory T cells. SLPI- and OVA-immunized SJL/J mice were sacrificed before EAE onset on day 12 after induction of adoptive transfer EAE and cells were extracted from inguinal lymph nodes. Analogous to the *in vitro *experiment, mice immunized with SLPI-protein showed increased numbers of CD4+ FoxP3+ regulatory T cells in lymph nodes compared to OVA-immunized animals (Figure 5) providing further evidence that the neutralization of SLPI increases the generation of regulatory T cells.

On the other hand, SLPI-immunization did not cause any difference in the number of Th1 and Th17 cells in lymph nodes at the end of the EAE experiment presented in Figure 2A. SLPI-and OVA-immunized mice were sacrificed on day 37 after induction of adoptive transfer EAE, their lymph nodes extracted and stimulated *in vitro *with PMA, Ionomycin and Brefeldin A for intracellular cytokine staining. No differences in the number of Th1 and Th17 cells were found (Additional file 4: Figure S4).

## Discussion

MS is a disease of unknown etiology in which there is substantial clinical and pathological evidence of auto-immune dysfunction [16,17]. The disease activity in MS at any time point likely reflects the state of equilibrium between pro-inflammatory and anti-inflammatory signals. Systemic immune activation, the integrity of the blood-brain barrier, and inflammatory events within the brain and spinal cord all contribute to demyelination and axonal damage [20]. The role of proteases in MS pathogenesis is of interest because they have crucial regulatory roles in inflammatory processes through a number of different mechanisms. These include modification and induction of chemokines and cytokines, activation of cellular receptors, cleavage of adhesion molecules, activation of TLR4 and matrix metalloproteinases (MMPs) (reviewed in [21,22]). In CNS inflammation in particular, MMPs may contribute to inflammatory damage through disruption of the blood brain barrier [23] and by cleaving myelin proteins [24]. Further investigation and understanding of the role of proteases in MS is therefore important to enable development of therapeutic strategies that modulate CNS inflammation [25].

To further elucidate the role of proteases in MS we studied the effects of the protease inhibitor SLPI in EAE, the accepted disease model of MS. SLPI is of interest because in addition to its competitive inhibition of several proteases [6], it is upregulated in EAE [3] and has both anti-and pro-inflammatory on the immune system. SLPI exerts anti-inflammatory effects on macrophages, neutrophils [9] and B cells [10]. It also regulates inflammatory responses by reducing inflammatory gene expression and diminishing inflammatory cell accumulation following tissue injury [11,26]. However, mice deficient in SLPI have increased TGF-β activity suggesting a pro-inflammatory SLPI function [13]. Moreover, SLPI suppresses TGF-β [14] and reduces the differentiation of human regulatory T cells induced by elastase [15].

We examined the role of SLPI in two different EAE models, namely, the PLP-induced EAE of SJL/J mice and the MOG-induced EAE in DA rats. While the former is a versatile model of relapsing T-cell mediated CNS inflammation [27], the latter, with features such as relapses, chronicity, extensive demyelination and contribution of both cellular and antibody-mediated effects, more closely resembles MS [28].

In order to study the role of SLPI within these animal models, we induced SLPI-specific antibodies by immunizing animals with recombinant SLPI protein together with the adjuvant aluminum hydroxide. Control animals were vaccinated with ovalbumin peptide and adjuvant. We cannot completely exclude that SLPI protein differs in its immunogenicity from ovalbumin peptide resulting in an unspecific modulation of the animals' immune system, but we did not find a difference in the number of Th1 or Th17 in the lymph nodes of SLPI- and OVA-immunized mice during EAE. Moreover, we waited six to eight weeks after the second SLPI vaccination before inducing EAE in order to reduce the nonspecific effects of the immunization procedure on EAE pathogenesis. We tested the antibodies' capacity to neutralize SLPI *in vitro*. Only IgG isolated from SLPI-immunized but not from control-vaccinated animals prevented SLPI's inhibition of LPS-induced IL-8 induction [9]. This data strongly suggests that we were able to induce SLPI-specific antibodies which block SLPI mediated effects and this effect was not a function of a generalized immune activation.

Despite the well-established influence of SLPI on immune reactions, this is the first report showing that SLPI influences inflammatory CNS diseases. In our experiments, mice which received recombinant SLPI for the first sixteen days after EAE induction developed a more severe disease course than control injected mice. After day 20 the two treatment groups had a similar disease severity, probably due to the limited half-life of SLPI [19] and to the induction of SLPI-specific antibodies *in vivo*.

On the other hand, the neutralization of SLPI by generating functional anti-SLPI antibodies induced by SLPI protein immunization of SJL/J mice and DA rats resulted in decreased EAE disease severity in both MS models. We speculate accordingly that the protection mediated by SLPI antibodies is a general phenomenon which is independent of the myelin antigen used for EAE induction as well as of the procedure to induce EAE.

The milder disease course in SLPI-immunized rats and mice was paralleled by increased sera levels of TGF-β compared to OVA-immunized animals. These data suggest that in EAE, the pro-inflammatory functions of SLPI probably associated with down regulation of TGF-β described above have a greater impact than SLPI's anti-inflammatory effects. TGF-β controls proliferation and differentiation of a number of cell types involved in acquired and innate immunity (reviewed in [29]). In particular reference to MS, TGF-β upregulates CD25+ Foxp3+ regulatory T-cells and systemic administration of TGF-β is associated with amelioration of inflammatory activity in EAE [30].

In our studies the mechanism of amelioration of disease severity in EAE associated with anti-SLPI antibodies is not established, but we hypothesized that it is in part due to the increased TGF-β serum levels which may enhance the generation of anti-inflammatory CD25+, Foxp3+ regulatory T-cells. Indeed in our EAE disease mouse model we found an increased number of CD25^hi^, Foxp3+ regulatory T-cells in lymph nodes compared the numbers seen in control animals. Furthermore, SLPI suppresses the expression of TGF-β in monocytic U937 cells, and mitogenically stimulated naïve human CD4+ T cell cultures derived from healthy donors contained significantly fewer regulatory T cells when incubated with SLPI protein. Additionally, SLPI treated T cell cultures showed a decreased ability to regulate the proliferation of CFSE labeled CD4+ T cells compared to results with cultures not containing SLPI. Supplementation of very small amounts of active TGF-β neutralized SLPI's inhibitory impact on the generation of regulatory T cells completely.

The mechanism by which SLPI regulates the expression of TGF-β-remains to be elucidated. SLPI's ability to inhibit the activation of NFκB may contribute to TGF-β, as the activation of NFκB induces TGF-β [31]. Immature dendritic cells exposed to elastase secrete higher amounts of TGF-β [15]. Hence, the inhibition of elastase activity by SLPI [7] may also contribute to the observed suppression of TGF-β by SLPI in our studies.

These experiments support our hypothesis that the neutralization of SLPI is protective in adoptive transfer and active EAE probably because of an increase of TGF-β serum level promoting the differentiation of regulatory T cells. In previous work we had shown increased spinal expression of SLPI during MOG-induced EAE of DA rats and had speculated that SLPI was protective in the disease model because of its anti-inflammatory functions [3]. Our current experiments, however, suggest that the neutralization of SLPI effects in EAE leads to elevated TGF-β serum levels and is disease protective. Thus, the increased expression of SLPI in EAE in our previous work is likely a deleterious factor counteracting the TGF-β-mediated regulatory immune responses in the CNS.

## Conclusions

The influence of SLPI on CNS autoimmunity was not addressed before. Despite SLPI's manifold anti-inflammatory properties, the severity of both, rat murine EAE, was reduced by the induction of neutralizing SLPI antibodies. We provide evidence that SLPI exerts pro-inflammatory functions by interference with the production and/or activation of the anti-inflammatory molecule TGF-β. Further studies are warranted to define SLPI's role within CNS autoimmunity.

## Competing interests

The authors declare that they have no competing interests.

## Authors' contributions

AMM conceived the design of the study, performed experiments as well as experimental analysis and prepared the manuscript. EJ and HC provided technical support for the experiments and for data collection and drafted the manuscript. SAS provided guidance for all aspects of the work. All authors read and approved the final version of the manuscript.

## Supplementary Material

Additional file 1 **Figure 1**. SLPI immunization of DA rats induces SLPI specific antibodies. Rat sera collected 14 days after the second vaccination with SLPI- or OVA-protein were analyzed by ELISA for the presence of SLPI-specific antibodies, as described in Materials and Methods. Results represent the optical density at 405 nm (OD405). IgG titers represent the average (Av) titers of six different rats. Click here for file

Additional file 2 **Figure 2. SLPI administration increases severity of active PLP-induced EAE of SJL/J mice**. A: Female SJL/J mice were immunized with PLP-peptide. Between 0 and 16 days, SLPI mice were injected three times a day with recombinant SLPI (100 μL of 3.3 μg/mL in 0.9% saline); control mice were injected with 0.9% saline instead. Data is shown as mean ± S.E. of the mean. The differences between the SLPI and the control group were statistically significant on day 19 after disease induction (Mann-Whitney rank sum test, p < 0.05). B: Mouse sera collected 30 days after EAE induction were analyzed by ELISA for the presence of SLPI-specific antibodies, as described in Materials and Methods. IgG titers representing the average of samples from five different mice were compared Click here for file

Additional file 3 **Figure 3**. SLPI treatment of human naïve CD4+ T cell culture does not influence differentiation of Th1 and Th17 cells. A: Human naïve CD4+ T cells were stimulated with anti-CD3- and anti-CD28-coated beads in RPMI medium in the absence or presence of 500 ng/mL SLPI. After four days, the cells were activated with PMA, Ionomycin and Brefeldin A for 4 h and analyzed by flow cytometry for CD4 (DAPI channel), IFN-g (Pe-Cy7 channel) and IL-17 (FITC channel). Left side: Representative dot blot of control treated naïve human CD4+ T cell cultures, right side: representative dot blot of T cell cultures incubated with 500 ng/mL SLPI. B: The column chart shows the average concentration of IFNg + CD4+ T cells in the CD4+ T cell cultures. p = 0.11 comparing numbers of IFNg + CD4+ T cells incubated with 500 ng/ml SLPI and without SLPI. A respective diagram showing the average number of Th17 cells is not shown due to the lack of these cells in our T cell cultures. Click here for file

Additional file 4 **Figure 4**. SLPI immunization does not modulate Th1 and Th17 immune responses. OVA- or SLPI-immunized SJL/J were sacrificed 37 days after passive EAE induction. LNC were extracted, *in vitro *restimulated with PMA, Ionomycin and Brefeldin A and analyzed by flow cytometry for CD4 (DAPI channel), IFN-g (APC channel) and IL-17 (PE channel). Mice immunized with SLPI protein showed no significant difference to OVA-immunized mice with regards to the number of Th1 and Th17 cells. Click here for file
