# Supplementary figures and images for: Inhibition of SLPI ameliorates disease activity in experimental autoimmune encephalomyelitis
Source: BMC Neurosci. 2012 Mar 21;13:30. doi: 10.1186/1471-2202-13-30 (PMC3352067; doi:10.1186/1471-2202-13-30)

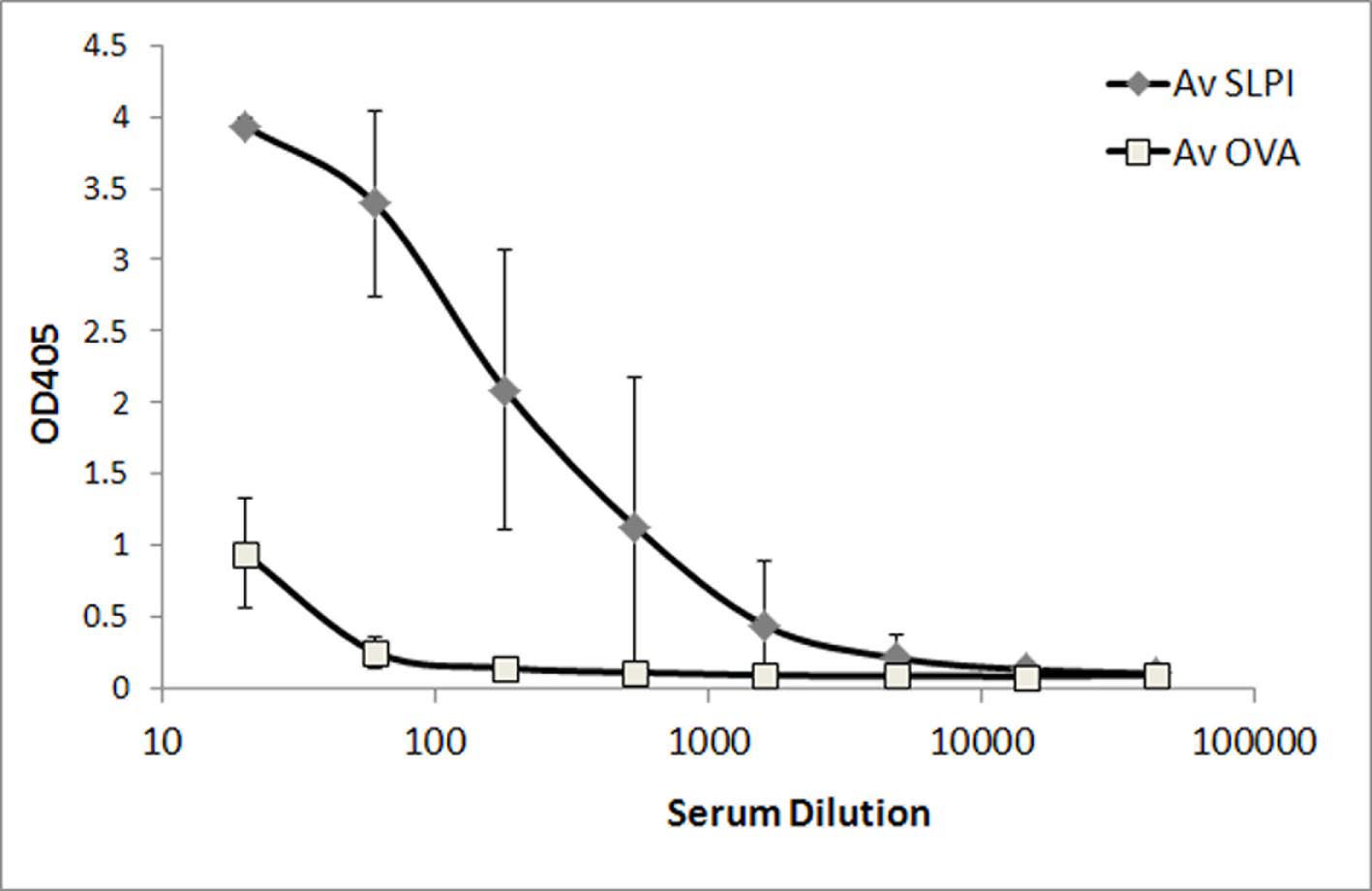

Supplement: Additional file 1 — Figure 1. SLPI immunization of DA rats induces SLPI specific antibodies. Rat sera collected 14 days after the second vaccination with SLPI- or OVA-protein were analyzed by ELISA for the presence of SLPI-specific antibodies, as described in Materials and Methods. Results represent the optical density at 405 nm (OD405). IgG titers represent the average (Av) titers of six different rats. [file 1471-2202-13-30-S1.TIFF]

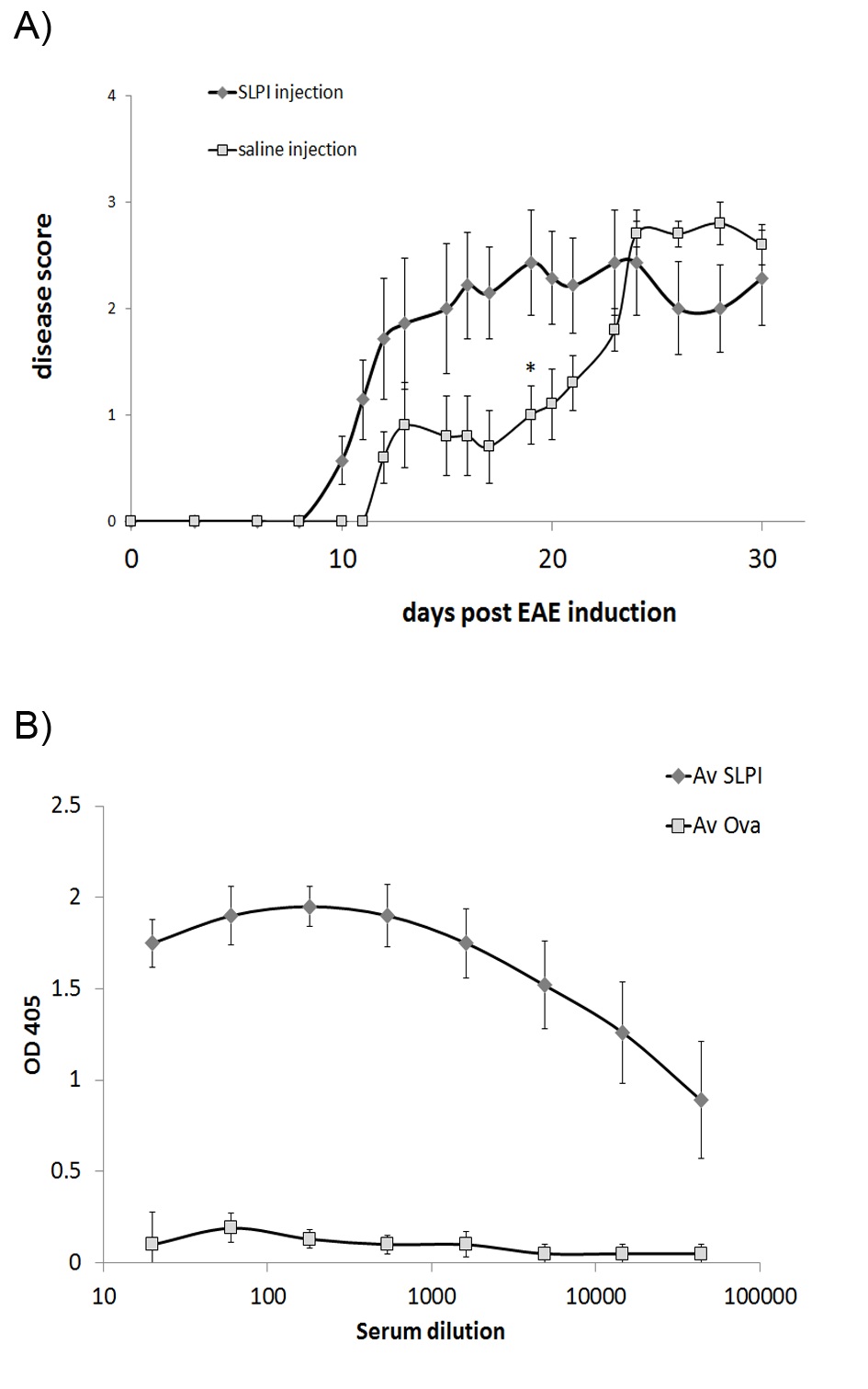

Supplement: Additional file 2 — Figure 2. SLPI administration increases severity of active PLP-induced EAE of SJL/J mice. A: Female SJL/J mice were immunized with PLP-peptide. Between 0 and 16 days, SLPI mice were injected three times a day with recombinant SLPI (100 μL of 3.3 μg/mL in 0.9% saline); control mice were injected with 0.9% saline instead. Data is shown as mean ± S.E. of the mean. The differences between the SLPI and the control group were statistically significant on day 19 after disease induction (Mann-Whitney rank sum test, p < 0.05). B: Mouse sera collected 30 days after EAE induction were analyzed by ELISA for the presence of SLPI-specific antibodies, as described in Materials and Methods. IgG titers representing the average of samples from five different mice were compared [file 1471-2202-13-30-S2.TIFF]

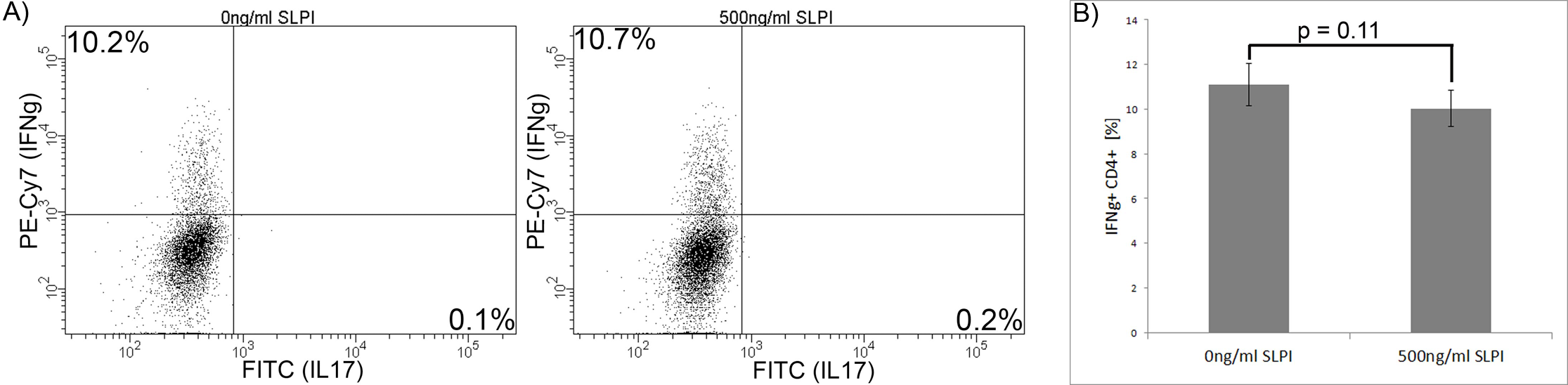

Supplement: Additional file 3 — Figure 3. SLPI treatment of human naïve CD4+ T cell culture does not influence differentiation of Th1 and Th17 cells. A: Human naïve CD4+ T cells were stimulated with anti-CD3- and anti-CD28-coated beads in RPMI medium in the absence or presence of 500 ng/mL SLPI. After four days, the cells were activated with PMA, Ionomycin and Brefeldin A for 4 h and analyzed by flow cytometry for CD4 (DAPI channel), IFN-g (Pe-Cy7 channel) and IL-17 (FITC channel). Left side: Representative dot blot of control treated naïve human CD4+ T cell cultures, right side: representative dot blot of T cell cultures incubated with 500 ng/mL SLPI. B: The column chart shows the average concentration of IFNg + CD4+ T cells in the CD4+ T cell cultures. p = 0.11 comparing numbers of IFNg + CD4+ T cells incubated with 500 ng/ml SLPI and without SLPI. A respective diagram showing the average number of Th17 cells is not shown due to the lack of these cells in our T cell cultures. [file 1471-2202-13-30-S3.TIFF]

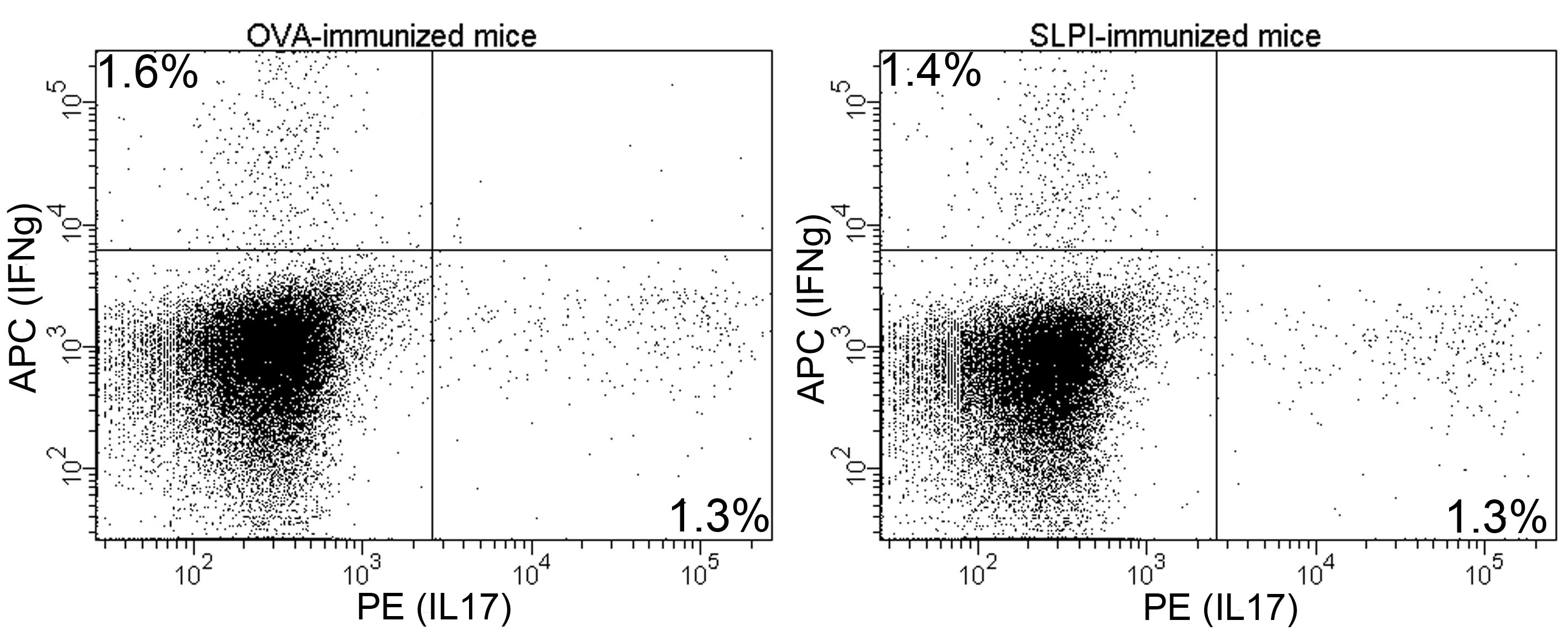

Supplement: Additional file 4 — Figure 4. SLPI immunization does not modulate Th1 and Th17 immune responses. OVA- or SLPI-immunized SJL/J were sacrificed 37 days after passive EAE induction. LNC were extracted, in vitro restimulated with PMA, Ionomycin and Brefeldin A and analyzed by flow cytometry for CD4 (DAPI channel), IFN-g (APC channel) and IL-17 (PE channel). Mice immunized with SLPI protein showed no significant difference to OVA-immunized mice with regards to the number of Th1 and Th17 cells. [file 1471-2202-13-30-S4.TIFF]
